# The Association of Specific Constituents of the Fecal Microbiota with Immune-Mediated Brain Disease in Dogs

**DOI:** 10.1371/journal.pone.0170589

**Published:** 2017-01-26

**Authors:** Nick D. Jeffery, Andrew K. Barker, Cody J. Alcott, Jon M. Levine, Ilyssa Meren, Jane Wengert, Albert E. Jergens, Jan S. Suchodolski

**Affiliations:** 1 Department of Veterinary Clinical Studies, College of Veterinary Medicine, Iowa State University, Ames, Iowa, United State of America; 2 Department of Small Animal Clinical Sciences, College of Veterinary Medicine & Biomedical Sciences, Texas A&M University, College Station, Texas, United State of America; University of Illinois at Urbana-Champaign, UNITED STATES

## Abstract

Meningoencephalomyelitis of unknown origin (MUO) is a common, naturally-occurring, clinical disease of pet dogs. It is an immune-mediated condition that has many similarities with experimental autoimmune encephalitis (EAE) in rodents and so investigation of its pathogenesis may aid in understanding factors that contribute to development of multiple sclerosis in people. Gut microbiota are known to modulate immune responses that influence susceptibility to immune-mediated brain disease. In this study we aimed to compare abundance of specific constituents of the fecal microbiota, namely *Faecalibacterium prausnitzii* and *Prevotellaceae*, between dogs diagnosed with MUO and matched controls. Fecal samples were obtained from 20 dogs diagnosed with MUO and 20 control dogs matched for breed, age and gender. Bacterial abundance was measured using qPCR and 16S rRNA sequencing. We found that *Prevotellaceae* were significantly less abundant in cases compared with controls (*p* = 0.003) but there was no difference in abundance of *F*.*prausnitzii*. There was no evidence of other differences in gut microbiota between groups. These data, derived from this naturally-occurring canine clinical model, provide strong corroborative evidence that high abundance of *Prevotellaceae* in the gut is associated with reduced risk for developing immune-mediated brain disease.

## Introduction

Disease in pet dogs treated by veterinarians forms a unique, frequently-overlooked, resource of biomedical translational data [1]. There are many advantages of these diseases as models of their human equivalents. First, they arise spontaneously, often through multifactorial etiologies similar to those that induce the parallel diseases in humans. Second, both pet dogs and humans share a common environment that may be a source of disease-causing risks. Third, diagnostic procedures and therapeutic interventions are broadly similar between humans and dogs with similar diseases. Within the field of neurology these features of similarity have been most extensively discussed in regard to spinal cord injury [2,3]. In this study we investigate possible etiologies for another canine disease: meningoencephalomyelitis of unknown origin (MUO), which models many aspects of multiple sclerosis in people.

MUO is an umbrella term used to summarize clinically-diagnosed immune-mediated inflammatory disease of the meninges, spinal cord and, especially, the brain of dogs [4]. On histopathologic examination a variety of sub-types of MUO are recognized, differentiated mainly by the predominance of white or grey matter involvement, the extent of necrosis and whether meninges are also inflamed [5,6], but all sub-types show the common feature of inflammatory cell infiltrate. In common with multiple sclerosis (MS) [7], much effort has been directed toward searching for infectious causes for MUO in dogs [8], and has proved similarly unsuccessful.

Traditionally, experimental autoimmune encephalomyelitis (EAE) in rodents has been widely used as a model for investigating the pathogenesis of multiple sclerosis [9]. The self-directed immune response in EAE can be initiated by injection of central nervous system (CNS)-derived antigens into susceptible animals or by passive transfer of antibodies from affected animals [10,11]. Recent work has suggested that the self-directed immune response, which is dependent upon a suitable systemic immune environment, can in turn be modulated by components of the gut microbiome [12]. Thus, germ-free mice are resistant to development of EAE, whereas those with wild-type gut microbiota remain susceptible [12,13]. Further support for this pathogenetic mechanism is provided by the changes in susceptibility to EAE that can be elicited by using antibiotics [14], oral immunization with vaccine strain of *Salmonella* [15], or probiotic mixtures [16].

Subsequent comparisons of the gut microbiome between MS patients and unaffected individuals have provided evidence that it may also play a role in the etiopathogenesis of MS [17,18]. Notably, an association has been made between development of MS and depleted populations of *Faecalibacterium prausnitzii*, *Prevotella* spp, or both [18], which also supports a more widely held belief that these two bacterial types may be protective against development of self-directed immune responses [19–21].

MUO in dogs shows many histological and immunological similarities to both EAE in rodents and MS in humans [22,23], suggesting a common etiology and implying its value in interpretation of the potential importance of the gut microbiome in development of immune-mediated encephalomyelitis in humans. Of further benefit is the low level of genetic diversity within defined dog breeds [24], implying that genetic influences on susceptibility to this disease [25–28] can be controlled by experimental design. In this study, we exploited the well-known breed, age and gender susceptibility [29] to design a case-control study in which to dissect the effects of gut microbial populations and other possible environmental triggers on development of MUO in dogs. We hypothesized that, in fecal samples obtained from dogs with MUO, there would be a decreased abundance of *F*.*prausnitzii* and, or, *Prevotellacea*, compared with that found in unaffected dogs.

## Methods

### Materials and methods

Fecal samples were collected from dogs (‘*Cases*’) diagnosed with MUO at the veterinary hospitals of Iowa State University (ISU) and Texas A&M University (TAMU) and stored in a -80°C freezer for analysis at completion of case recruitment. We also collected fecal samples from matched control dogs (‘*Controls*’) that were presented for diagnosis and treatment through the same veterinary school hospitals for other conditions.

#### Cases

Typical cases of MUO present with signs of brain dysfunction such as seizures or brainstem dysfunction (often disorders of vestibular function), although signs of disease anywhere in the CNS can be caused by MUO. Diagnostic tests include magnetic resonance imaging (MRI) scans and cerebrospinal fluid (CSF) analysis. Important rule-outs include infectious causes of brain inflammation such as *Toxoplasma*, canine distemper virus infection, *Neospora* and, depending on geographical location, tick-borne diseases. Final confirmation of the diagnosis is achieved through post mortem examination but cases can usually be securely diagnosed through clinical tests and long-term response to therapy alone.

**Fig 1:**
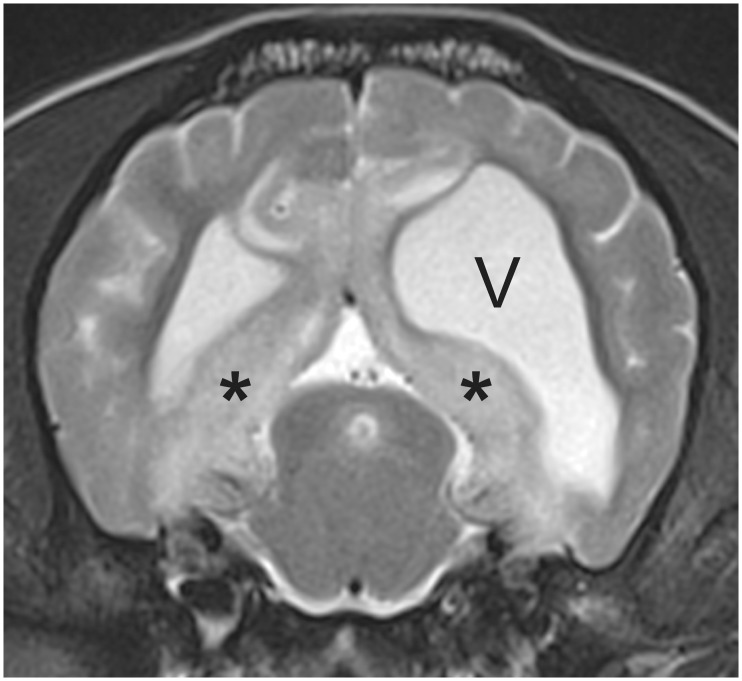
3T T2W axial MR image at the level of the caudal colliculus illustrating the typical hyperintensity (*) that is often prominent adjacent to the ventricles in cases of MUO. This dog also exhibits ventricular asymmetry (‘V’ indicates left ventricle), which is a common incidental finding in small breed dogs (this was a pug).

Potential *Cases* presented with various neurological signs relating to dysfunction of the brain (we specifically excluded dogs that were suspected of having MUO but had signs affecting the spinal cord or optic nerves alone), underwent routine MRI scanning and CSF analysis. For inclusion as a *Case* a dog had to show typical areas of diffuse hyperintensity on T2-weighted MRI scans (Fig 1) and increased cell counts (>5 white cells per μL) on CSF analysis. We also included any dogs that were definitively diagnosed at post mortem whether they fulfilled other clinical criteria or not. Dogs with evidence of gastrointestinal disease were specifically excluded, as were those that tested positive for any infectious disease. Any dog that had received oral antibiotics within 4 weeks of diagnosis, or that was diagnosed with cancer or immune-mediated disease affecting any part of the body, was also excluded.

After MRI and CSF had been obtained owners were asked for permission to take a fecal sample and for their assistance in completing the study record form to complete a short questionnaire (see below). This protocol was specifically reviewed and approved by the Iowa State University Institutional Animal Care and Use Committee (log number 4-12-7347-K).

#### Controls

These were dogs that presented to ISU or TAMU for investigation or treatment of other conditions but were of the same breed, gender and matched age. In line with recommendations for case-control studies [30], we specifically sought *Control* animals from those presented to our veterinary university clinics because they were derived from the same population as the *Cases*. *Contro*l dogs were subject to the same exclusions as *Cases*, therefore dogs diagnosed with any gastro-intestinal disease, proven or suspected immune-mediated disease, cancer, or that had received systemic antibiotic therapy within 4 weeks were excluded. Owners of dogs that fulfilled these requirements (identified through daily appointment records) or that were of an age and breed likely to match in future with a *Case* were approached to seek consent to obtain a fecal sample and complete the *Study Record Form*, whether or not a matched *Case* had previously been recruited.

#### Fecal samples

Feces (at least 30g) were obtained from the rectum using a gloved finger and then stored in sterile containers in a -80°C freezer until the end of the sample acquisition phase.

#### Study Record Form

A specifically-designed *Study Record Form* recorded demographic data plus whether the dog was a *Case* or *Control* and the date of the most recent antecedent vaccination (of any type). Vaccination of both *Cases* and *Controls* within the previous calendar month was recorded as a dichotomous variable. The home environment in which the dog lived was categorized as rural, semi-rural (small town) or urban. We also asked questions about diet in general terms, such as whether dry (kibble), wet (canned) or home-cooked food was provided but did not request details. Any further follow-up information (*e*.*g*. post-mortem report) was added as free text.

#### Gut microbiota analysis

DNA isolation: DNA was extracted from the swabs with a MoBio Power soil DNA isolation kit (MoBio Laboratories, USA) following the manufacturer’s instructions.

Sequencing of 16S rRNA genes: The V4 region of the 16S rRNA gene was amplified with primers 515F (5’-GTGCCAGCMGCCGCGGTAA-3’) and 806R (5’-GGACTACVSGGGTATCTAAT-3’) at the MR DNA Laboratory (Shallowater, TX, USA) as previously described [31]. The Nextera^®^ DNA sample Preparation kit including sequencing adapters and sample specific barcodes was used to prepare a DNA library and sequenced at MR DNA on an Illumina MiSeq instrument.

The raw sequence data was screened, trimmed, filtered, denoised and barcodes and chimera sequences were depleted from the dataset using QIIME v1.8 pipeline and UCHIME. Operational Taxonomic Units (OTUs) were assigned based on at least 97% sequence similarity against the Greengenes reference database. Sequences were rarefied to an even depth of 6,900 sequences per sample to account for unequal sequencing depth across samples. Observed species richness, Chao 1, and Shannon indexes were determined using QIIME. The sequences were deposited in SRA under accession number PRJNA319388.

#### Statistical analysis

Our study was designed to be analyzed using conditional logistic regression so as to take advantage of the case-control design, in which each *Case* was matched with a single *Control* of the same breed, gender and approximate age. MUO status (yes or no) was the dependent outcome variable. The primary, pre-defined outcome measure—based on the previous data on association in multiple sclerosis [18]—was to test whether reduced abundance of *F*.*prausnitzii* and *Prevotellaceae* was significantly associated with diagnosis of MUO. We also examined the association with two other putative risk factors for MUO that might also have relevance for development of MS in people: vaccination status (vaccinated within the previous month or not) and environment (in three graded categories). Analysis was conducted using Stata 11.0 (Stata Corp, College Station, TX) with P<0.05 taken to indicate significance.

We also planned further exploratory comparison of the microbial populations between *Cases* and *Controls* using analysis of beta-diversity by unweighted Unifrac distance metrics, similarly to a previously published report [31]. Statistical significance of the resulting distance metric was tested by analysis of similarities (ANOSIM) using the QIIME software.

There was no formal power calculation for this study, because we had little previous available information on which to base a sample size calculation, although the study by Miyake et al (2014) [18] examined 20 human MS patients. Instead, we aimed to accumulate as many matched pairs as possible during the 2-year period of study sponsorship (American Kennel Club Health Foundation, grant # 01731).

## Results

Samples with adequate documentation of all variables were collected from a total of 70 dogs, of which 39 were *Cases* and 31 were *Controls*. As expected from previous studies [29], the majority were small breed dogs (<20kg bodyweight). There were 20 matched pairs of dogs (40 dogs in total); 5 pairs were male and 15 pairs were female; this gender predominance is also similar to that reported in previous studies [29].

The full breed list is: Chihuahua (n = 8), Maltese (n = 6), Labrador (n = 4), pug (n = 4), shih-tzu (n = 4), then golden retriever, Chesapeake bay retriever, Weimeraner, dachshund, beagle, miniature pinscher and bichon frise (each n = 2). *Cases* were presented at the following periods in the year: 8 dogs in winter (Dec-Feb); 6 dogs in spring (Mar-May); 3 dogs in summer (Jun-Aug) and 3 dogs in fall (Sep-Nov).

### Pre-specified comparisons between Cases and Controls

#### Univariable analysis

**Fig 2:**
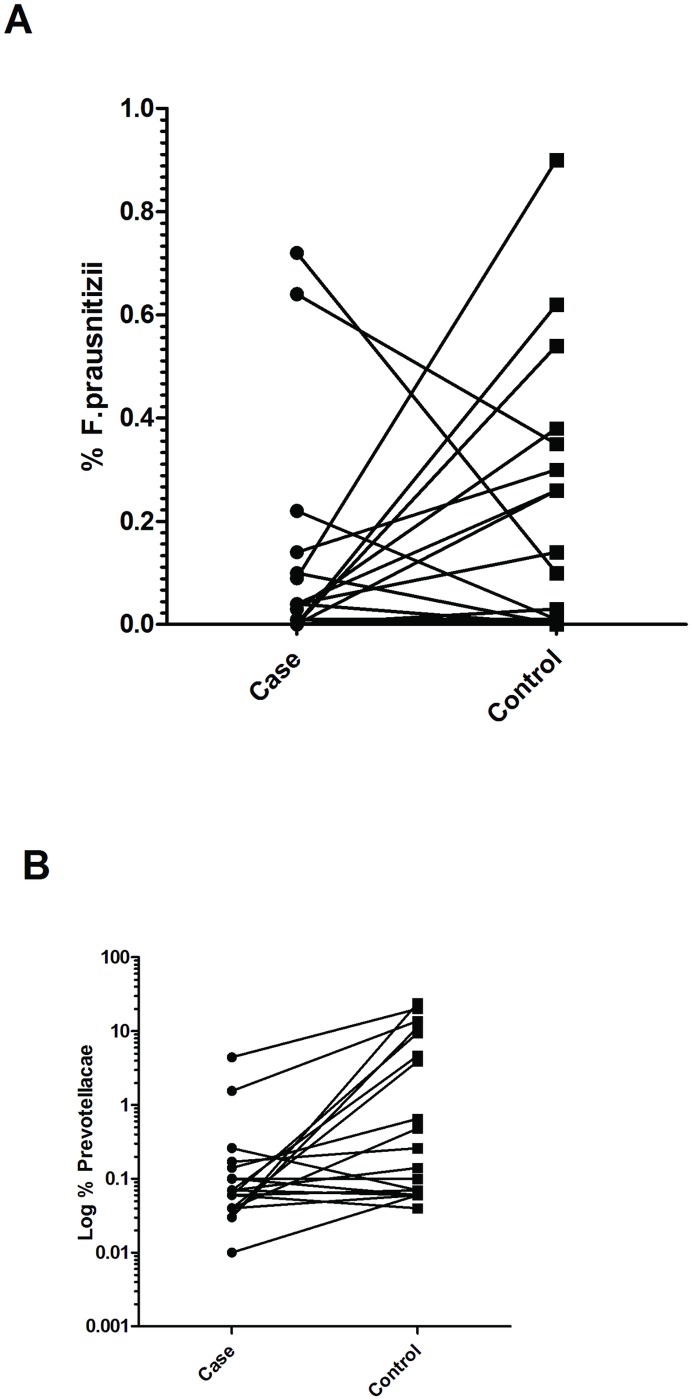
Dot plots illustrating relative abundance of *F*.*prausnitzii* (A) and *Prevotellaceae* (x10^3^) (B) in Cases and matched Controls. *Prevotellaceae* abundance is shown on a log scale to improve detail; this is not possible for *F*.*prausnitzii* because many animals had a zero score.

Initially we examined the abundance of *F*.*prausnitzii* and *Prevotellaceae* in *Cases* and their matched *Controls*, neither of which constituted normally-distributed data. Amongst all sampled dogs *F*. *prausnitzii* abundance ranged from 0–0.9% of the total bacterial population and differed little between *Cases* and *Controls* (Wilcoxon paired signed ranks test, P = 0.198). In 9 of 21 *Cases* and 5 of 21 *Controls* no bacteria of this species were detected at all. Abundance of *Prevotellaceae* ranged from 0.01–23.91% across all sampled dogs but was significantly different between *Cases* and *Controls* (Wilcoxon paired signed ranks test, P = 0.003) (Fig 2). The data we recorded on diet proved to be insufficiently detailed to allow further analysis. Owners in both groups reported feeding similar diets in general terms: 17/21 *Cases* and 19/21 *Controls* were fed various brands of kibble food as their main, or only, diet. However, most owners also indicated that there was variation in the daily diet and many dogs received variable amounts of table scraps and rewards.

Conditional logistic regression (which takes advantage of pair-matching) was used to examine the relationships between *Prevotellaceae* and *F*.*prausnitzii* abundances and development of MUO in more detail. Because the data populations were not normally-distributed and because we could not assume that relationships between bacterial abundance and development of MUO were linear we divided the populations into tertiles (*i*.*e*. low, medium and high abundance) using an automatic function in Stata. This analysis revealed a tendency toward lower prevalence of MUO in animals with higher abundance of *F*.*prausnitzii*, although this was not statistically significant in this population (Odds ratio [OR] = 0.575; P = 0.192). In contrast there was a stronger, statistically-significant relationship of reduced likelihood of MUO in dogs with higher abundance of *Prevotellaceae* (OR = 0.303; P = 0.038) (Table 1). This was also reflected in the significant trend of decreasing odds of developing MUO with increasing abundance of *Prevotellaceae* (χ^2^ = 6.89; P = 0.010) (Table 2).

**Table 1:** Results of univariable conditional logistic regression analyzing association of various putative risk factors (expressed as categories) with the development of MUO.

	Odds ratio	SE	Z	P	95% CI
**F.prausnitizii**	0.574	0.244	-1.30	0.192	0.250–1.322
**Prevotellaceae**	0.303	0.175	-2.07	0.038	0.098–0.937
**Environment**	2.809	1.584	1.83	0.067	0.930–8.481

**Table 2:** Analysis of odds of development of MUO associated with various abundances of *Prevotellaceae*.

Prevotellaceae abundance	Cases	Controls	Odds	95% CI
**Low**	7	1	7.000	0.861–56.895
**Medium**	9	9	1.000	0.397–2.519
**High**	4	10	0.400	0.125–1.275

Test of trend of odds: χ^2^ = 6.65; P = 0.010

We next examined the possible effects of two other possible environmental factors: recent vaccination and home environment. None of the unaffected *Control* dogs had been vaccinated within the past month, whereas 5/20 *Cases* had been, resulting in a statistically significant association (Fisher’s exact test, p = 0.047). Living environment appeared to be weakly associated with development of MUO; overall the odds of developing MUO were lower for dogs living in a rural environment that for those living in urban areas although not significant at the P<0.05 level (OR = 2.810; P = 0.067; Table 1) and this was also reflected in the significant trend of odds associated with increasingly more populated environments (χ^2^ = 4.53; P = 0.033; Table 3).

**Table 3:** Analysis of odds of development of MUO associated with various environmental conditions.

Environment	Cases	Controls	Odds	95% CI
**Rural**	1	6	0.167	0.020–1.384
**Small town / edge of city**	8	8	1.000	0.375–2.664
**Urban**	11	6	1.833	0.678–4.957

Test of trend of odds: χ^2^ = 4.53; P = 0.033

#### Multivariable analysis

We intended to include all the relevant variables in multivariable conditional logistic regression but, because of the lack of recent vaccination in any *Control* dogs, it was not possible to include this variable. When all three other variables were included together in multivariable conditional regression analysis there was considerable change in the association of diagnosis of MUO with the dog’s living environment (OR = 10.936), and moderate changes for association with *F*.*prausnitzii* (OR = 0.255) and with *Prevotellaceae* (OR = 0.446) (Table 4). A series of subsequent exploratory analyses using stratification of exposure to both *Prevotellaceae* and environmental categories, plus χ^2^ analysis of association between these variables, were impaired by null values in some categories. Nevertheless, inspection of the odds ratios (and the instability of the figures for environment) strongly suggests interaction amongst these variables.

**Table 4:** Multivariable conditional logistic regression analyzing association of various putative risk factors with development of MUO.

	Odds ratio	SE	Z	P	95% CI
**F.prausnitizii**	0.255	0.227	-1.54	0.125	0.044–1.460
**Prevotellaceae**	0.446	0.291	-1.24	0.216	0.124–1.603
**Environment**	10.936	16.051	1.63	0.103	0.616–194.142

#### Exploratory analysis of bacterial phyla populations

One of the difficulties in analyzing gut microbiota is that there is an almost infinite number of possible hypotheses to test. Nevertheless, our next step was preliminary exploratory analysis of the bacterial phyla abundances that were available from the sequencing studies. Similar studies have been conducted for human patients with MS [17,18] and we used a parallel methodology.

**Fig 3:**
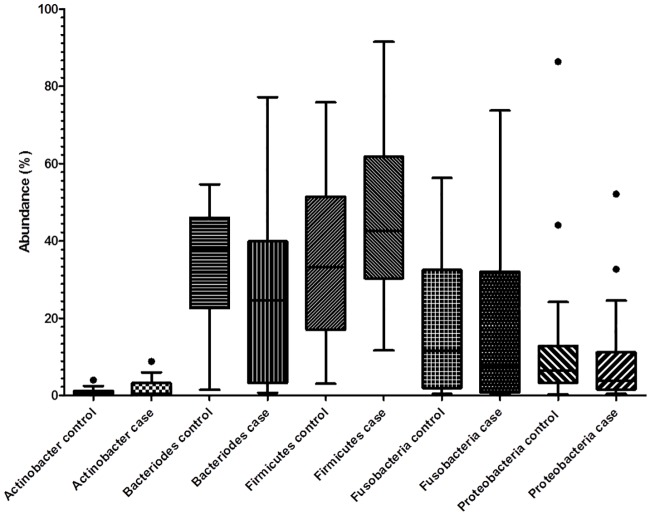
Tukey box-and-whisker plots illustrating relative abundance of the major bacterial phyla in fecal samples from *Case* and *Control* dogs.

Abundance of bacteria was recorded for each of 8 major phyla (*Euryarchaeota*, *Actinobacteria*, *Bacteroidetes*, *Deferribacteres*, *Firmicutes*, *Fusobacteria*, *Proteobacteria*, *Tenericutes*). Abundance was recorded as zero in a substantial proportion (>50%) of all dogs in some categories (*Euryarchaeota*, *Deferribacteres*, *Tenericutes*) leaving a total of 5 phyla for analysis of the relationship between abundance and diagnosis of MUO. For none of these phyla was there evidence of a statistical difference in abundance between *Case*s and *Controls* (Fig 3) and conditional logistic regression analysis of the matched-pair data using tertiles (as described above for *Prevotellaceae* and *F*.*prausnitzii*) did not support an association between abundance of any of these bacterial phyla and development of MUO (Table 5).

**Table 5:** Results of exploratory multivariable conditional logistic regression on association of various bacteria phyla with development of MUO.

	Odds ratio	SE	Z	P	95% CI
**Actinobacteria**	1.168	0.514	0.35	0.724	0.493–2.768
**Bacterioidetes**	0.456	0.230	-1.55	0.120	0.170–1.228
**Firmicutes**	0.736	0.411	-0.55	0.583	0.247–2.197
**Fusobacteria**	0.705	0.371	-0.66	0.508	0.251–1.980
**Proteobacteria**	0.582	0.293	-1.07	0.283	0.217–1.563

**Fig 4:**
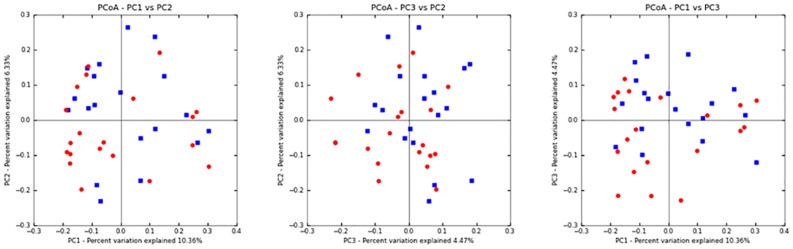
2-dimensional representations of the principal component analysis illustrating the lack of clustering of microbiota constituents in *Cases* (blue squares) or *Controls* (red dots).

Finally, we used principal component analysis as a means to detect more global association between fecal microbial content and development of MUO. Alpha diversity as described by Chao 1, Observed species (species richness), and Shannon diversity index were not significantly different between paired samples. PCoA plots of unweighted Unifrac distances (Fig 4) confirmed that there was not significant clustering between samples from *Case* or *Control* groups (R-statistic = 0.054; ANOSIM p = 0.15).

## Discussion

The analysis presented here supports an association between low *Prevotellaceae* abundance and a diagnosis of MUO in dogs, which also corroborates previous observations of an association between this bacterial group and diagnosis of MS in people [18]. Such corroboration, especially in a different species, is important because investigation of such a complicated system as the gut microbiome carries an inherently high risk of false discovery. In this study, we specifically targeted only two bacterial populations in our primary pre-specified analysis implying that the risk of false discovery is low. Therefore, this evidence carries high value as corroboration of the importance of *Prevotellaceae* in reducing risk of immune-mediated CNS disease. Interestingly, low abundance of *Prevotellaceae* has also been associated with Parkinson’s disease in people [32]. The rigid case-control design has enabled us to minimize the possible confounding factors of age, gender and genetic variability.

On the other hand, the possible association of low abundance of *F*.*prausntizii* with MS in people [17,18] has not been supported by our data. This could simply be because of the relatively low power of our study, especially since many of the dogs had zero counts of this bacterium that may have confounded our ability to make associations. Nevertheless, our observations on matched pairs of dogs (Fig 1) would suggest that any effects must be relatively small since in many pairs the *Case* had a higher abundance of *F*.*prausnitzii* than its matched *Control*. It is also possible that for some reason, such as diet, dogs in general, or dogs in this specific study, simply have low levels of *F*.*prausnitzii* and its association with immune-mediated disease in this species is not the same as in others. Lastly, in the study by Canterel at el (2015) [17] the abundance of various bacterial groups, including *Faecalibacterium*, was altered by vitamin D levels. Because dogs are not dependent on sunlight for production of vitamin D [33], levels of this vitamin are likely adequate in dogs receiving a reputable manufacturer’s diet (as all dogs in this study were) and so the relationship between levels of this vitamin and abundance of this specific fecal bacterium may differ between the two species.

Two further findings in this study may be useful in research into etiology of MS in humans. First, we show the possibility that MUO may, in some cases, be triggered by vaccination. However, it is important that this finding be treated with caution: there were few dogs that had received vaccination and developed disease and so confidence in this result must be low. Nevertheless, the possibility of vaccine triggering MS or other CNS demyelinating conditions has been previously studied in MS patients; overall the conclusions have been that there is no evidence of increased risk [34,35], although there is a suggestion that some individuals that are in sub-clinical stage of other demyelinating diseases may be triggered into disease by vaccination [35]. Our results are consistent with this explanation.

Pet dogs can also have potential for helping to identify disease risk factors in the environment because they share living spaces with humans and so may have similar exposure patterns. In this study, there is evidence that an urban environment is associated with increased risk of MUO. Our study provides a little support for the notion that this risk is mediated via effects on the microbiome (because there was weak evidence of interaction between environment and *Prevotellaceae* abundance) and so other mechanisms could be evoked. In humans there is strong evidence that specific regions of the world carry risk for development of MS, in particular through linkage with vitamin D status [36]. This would be an unlikely association in dogs because, as noted above, they do not manufacture vitamin D in the skin [33]; instead, dogs may be able to provide evidence of more local environmental risk, such as the characteristics of a specific neighborhood that might play a role in susceptibility to immune-mediated CNS diseases. There is some previous evidence that urban living may be a risk factor for MS in people [37], and this has been suggested to be because of increased hygiene. It is possible that the same could apply to dogs living in urban environments (since they have less exposure to wildlife, for instance) but, alternatively, urban-living dogs may also have greater exposure to environmental toxins, such as organic solvents, which have been suggested as possible risks in human immune-mediated disease [38]. On the whole, dogs are excellent sentinel species for environmental toxins because of their well-developed scavenging behavior.

Finally, the association of high abundance of *Prevotellaceae* with reduced likelihood of developing MUO may have two general explanations. As a ‘fermenting’ group of bacteria, *Prevotellaceae* produce butyrate, which has been identified as a specific inducer of T_reg_ cell differentiation [39]. It is possible that this butyrate-producing metabolic profile may thus constitute the uniting mechanism of resistance to immune-mediated disease thought to be mediated by both *Prevotellaceae* and *F*. *prausnitzii* [21]. Similarly, hormones produced by specific gut microbes could also directly influence the immune system [40]. Together these effects may modulate the changed intestinal permeability that has been detected in both MS patients [41] and rats with EAE [42]. On the other hand, it is also possible that high *Prevotellaceae* abundance might result as a bystander effect of another interaction between the host (immune system) and the gut microbiome. If this were true, a high population of *Prevotellaceae* might simply be a biomarker of an immune system that is in a state that moderates inflammatory responses. Support for this notion is provided by their relatively low overall abundance: *Prevotellaceae* were recorded in our study as a median of only ~0.2% of the detected bacterial microbiota; nevertheless, hormonal effects need not depend on high abundance.

What limitations might there be in the conclusions that can be reached from this study? First, because it was designed with the specific aim of exploring the possibility that reduced fecal bacterial population of one pre-specified species and one pre-specified family might be associated with development of this specific immune-mediated disease these results must be regarded as robust. There is little risk of a false positive finding with this experimental design and, furthermore, the outcome largely corroborates previous evidence. Nevertheless, there are an almost unlimited number of unrecorded variables, such as specific dietary components, dog activity levels and gut permeability that might possibly have some influence on the development of MUO and might have been overlooked in this study. On the other hand, there is little reason to suppose that these factors would be systematically differently distributed between our *Case* population and the *Controls*, especially since we specifically selected our *Controls* from animals presenting to our clinics for treatment. Further investigation in new sample populations is required to investigate all the possible interactions amongst gut microbiota, environment and other disease states.

Finding different abundances of specific bacterial species between affected and unaffected groups in this study directs a spotlight at diet as a possible cause of these differences. Here we recorded only coarse information that proved impossible to analyze in a meaningful way since there was insufficient detail to be able to analyze the proportions of specific nutrients in any particular animal’s diet. Unfortunately, pet dog diets are often difficult to analyze because there is a great deal of variability in the way that owners feed dogs, both between individuals and between days. Further exploration of these dietary effects will require collaboration with a specialist nutritionist.

There is an almost infinite variety of environmental factors that could be involved in the risk of developing MUO and it is not possible for all these to be investigated, or accounted for, in a single study. For instance, there is some evidence that ingestion of non-steroidal anti-inflammatory drugs (NSAIDs) may alter not only gut permeability, but also may be associated with changes in gut microbiota [43]. Unfortunately, investigation of such effects is complicated because different NSAIDs appear to be associated with different gut microbial constituents and may also be affected by duration of therapy. Therefore, the possible effect of NSAID therapy, which was accounted for in this study as an unknown that would ‘randomize out’, provides an example of a factor that requires further investigation in the context of CNS inflammatory disease. On the other hand, in this study we did collect simple data on a small number of some possible explanatory variables, with the aim of providing some preliminary information on possible risk factors for development of MUO in dogs and, by implication, possibly also for MS in humans. The study design implies that the results of analysis of the association of these other factors (apart from the targeted bacterial populations) with development of MUO must be regarded as tentative and will require further validation in repeated studies. For instance, the apparent association of recent vaccination with development of MUO is weak—few dogs had been vaccinated in temporal proximity to disease onset and so the confidence intervals associated with this possible risk are wide—implying low analytical power and low confidence in their repeatability. Furthermore, it is plausible that different vaccinations might differ in their effects. The association of urban living with development of MUO is more robust, especially since there is a strong trend of odds across the range of exposure (Table 4) and there is a previous suggestion of a link between urban living and development of MS in people [37]. Nevertheless, our environmental data are not highly detailed (for instance we did not link zip code to population density) and will therefore require verification in a new sample population.

## Supporting Information

S1 Table Signalment, Prevotellacae and F prausnitzii abundance. https://dx.doi.org/10.6084/m9.figshare.4541227.v1. doi: 10.6084/m9.figshare.4541227.v1.
(XLSX)

S2 Table Summary of bacterial phyla data. https://dx.doi.org/10.6084/m9.figshare.4541233.v1. doi: 10.6084/m9.figshare.4541233.v1.
(XLSX)
